# Translating *Niphargus* barcodes from Switzerland into taxonomy with a description of two new species (Amphipoda, Niphargidae)

**DOI:** 10.3897/zookeys.760.24978

**Published:** 2018-05-28

**Authors:** Cene Fišer, Roman Alther, Valerija Zakšek, Špela Borko, Andreas Fuchs, Florian Altermatt

**Affiliations:** 1 SubBio Lab, Department of Biology, Biotechnical Faculty, University of Ljubljana, Večna pot 111, SI-1000 Ljubljana, Slovenia; 2 Eawag: Swiss Federal Institute of Aquatic Science and Technology, Department of Aquatic Ecology, Überlandstrasse 133, CH-8600 Dübendorf, Switzerland; 3 Institut für Grundwasserökologie IGÖ GmbH an der Universität Koblenz-Landau, Campus Landau Fortstraße 7, D-76829 Landau, Germany; 4 Department of Evolutionary Biology and Environmental Studies, University of Zurich, Winterthurerstr. 190, CH-8057 Zürich, Switzerland

**Keywords:** Amphipoda, barcodes, DELTA, groundwater, integrative taxonomy, Niphargidae, web-taxonomy

## Abstract

The amphipod genus *Niphargus* (Amphipoda: Niphargidae Bousfield, 1977) is the most species-rich genus of freshwater amphipods in the World. Species of this genus, which live almost exclusively in subterranean water, offer an interesting model system for basic and applied biodiversity science. Their use, however, is often limited due to the hitherto unresolved taxonomy within the whole genus. As a comprehensive taxonomic revision of the currently >425 *Niphargus* species is too demanding, it has been suggested that the taxonomy of the genus could be advanced in smaller steps, by reviewing regional faunas, that would eventually integrate into a global revision. In this study, we provide such a revision of *Niphargus* in Switzerland. First, we molecularly delimited, morphologically diagnosed, and formally described two new species, namely *Niphargus luchoffmanni* **sp. n.** and *Niphargus tonywhitteni* **sp. n.** Second, we updated and revised a checklist of *Niphargus* in Switzerland with new findings, and prepared a list of reference sequences for routine molecular identification, available at BOLD and GenBank. All available specimens of 22 known species from the area were morphologically examined, and their morphological variation was compiled in a data file of DEscription Language for TAxonomy, which can be used for automated generation of dichotomous or interactive keys. The data file is freely available at the World Amphipoda Database. Together, the checklist, the library of reference sequences, the DELTA file, but also a list of hitherto unresolved aspects are an important step towards a complete revision of the genus within a well-defined and biogeographically interesting area in Central Europe.

## Introduction

*Niphargus* Schiødte, 1849 (Amphipoda: Niphargidae Bousfield, 1977) is an amphipod genus living almost exclusively in groundwater ecosystems of the West Palearctic (Copilaş-Ciocianu et al. 2018). With >425 described species (Horton et al. 2018) it is among the most species-rich freshwater amphipod genera (Väinölä et al. 2008, Horton et al. 2018), and an important representative of European groundwater macroinvertebrate fauna (Zagmajster et al. 2014). The genus is an interesting model system for biogeography (McInerney et al. 2014), evolutionary ecology (Trontelj et al. 2012, Copilaş-Ciocianu et al. 2018), and applied ecology (Marmonier et al. 2013).

The use of *Niphargus* species in applied ecology is often limited due to the partly unresolved and still incomplete taxonomy within the genus. The taxonomic incompleteness in the first place mirrors the biology and ecology of *Niphargus*: many species have narrow ranges, sometimes spanning only a few kilometres around their type localities (Meleg et al. 2013) and ranges extending beyond a few hundred kilometres are the exception (Trontelj et al. 2009, Copilaș-Ciocianu et al. 2017, Copilaş-Ciocianu et al. 2018). Consequently, any newly investigated cave can potentially harbour new, undescribed species. Moreover, many species are elusive and can only be found after repeated sampling (Pipan and Culver 2007, Fišer and Zagmajster 2009). This often involves intense fieldwork on a fine scale, with difficulty to access habitats, and often requires advanced caving techniques and the help of local cavers. Second, the genus is characterised by inherent challenges with respect to species delimitations. Morphological differences between species are often subtle, while intraspecific variation can be high (Fišer et al. 2008, Fišer and Zagmajster 2009, Delić et al. 2016). A low number of specimens per sample often hampers further insights into intra- and interspecific variation, and limits taxonomic decisions based on morphology only. Consequently, taxonomic evaluations need to be complemented with molecular data, and in some species-complexes diagnoses entirely depend on diagnostic sequences (Delić et al. 2017a, b). With cryptic species (i.e., morphologically indistinguishable species) being repeatedly found in *Niphargus*, the revision of the genus is feasible only upon critical assessment of molecular, morphological, geographical and ecological data, that is, within an integrative taxonomy framework (Padial et al. 2010, Yeates et al. 2011).

Overall, a comprehensive taxonomic revision of *Niphargus*, encompassing all hitherto described species between Ireland and Iran, is technically challenging and unlikely to be completed in the near future. To make *Niphargus* accessible for various end-users of taxonomy, such as naturalists, ecotoxicological laboratories, or nature conservation agencies, local revisions rather than a single global revision represent a more realistic way forward. Such geographically restricted revisions could link local species checklists, diagnostic morphological traits and barcoding sequences, and thereby make the group accessible to these users. Local revisions can be completed within a realistic time, and also contain fewer morphologically similar species than an eventual global revision. In the long term, carefully composed local revisions, based on the inclusion of appropriate outgroups and representative species from the whole genus, can be integrated into a global revision.

The idea of geographically restricted revisions was already applied to *Niphargus* in the Middle East (Esmaeili-Rineh et al. 2015a). An initial overview of the fauna (Fišer et al. 2009a) was followed by field work. Samples from the Middle East were first delimited using molecular phylogenetic tools (Esmaeili-Rineh et al. 2015b), and then complemented by morphological analyses, morphological diagnoses and a construction of a morphological database using DEscription Language for TAxonomy (DELTA ; Esmaeili-Rineh et al. 2017b). The latter presented the basis for all subsequent morphological comparisons but also for automated constructions of interactive or dichotomous identification keys. Until now, this database has been continuously updated with descriptions of new species (Mamaghani-Shishvan et al. 2017, Esmaeili-Rineh et al. 2017a, Esmaeili-Rineh et al. 2017b).

A similar approach was applied to the geographically restricted diversity of *Niphargus* species in Switzerland. An initial checklist and molecular exploration (Altermatt et al. 2014) was complemented by further sampling and descriptions of new species (Fišer et al. 2017). The latter studies identified several species awaiting taxonomic evaluation. Here, we further advance this revision of *Niphargus* from Switzerland. We first described two more species. Second, in order to accelerate further research of groundwater communities in Switzerland, we overviewed current knowledge of the taxonomy of the genus and constructed a DELTA database of morphological characters. Finally, we prepared a reference library of COI sequences, and linked them to species names.

## Materials and methods

### Sampling and origin of specimens

The studied specimens derive from various sampling campaigns (2010–2017). Most of the specimens from the newly described species were sampled for a larger study on springs in the Swiss mountains (Verena Lubini and Aquabug AG, Neuchâtel, Switzerland). The sample from the Töss River interstitial was collected using a hand pump. The sample from Achensee in Austria was sampled using a rectangular kicknet (25 × 25 cm) with mesh size of 500 μm and disturbing the littoral zone manually. Specimens were conserved in ethanol. Most of the samples (67) were already analysed in previous studies (Altermatt et al. 2014, Fišer et al. 2017); in this study we molecularly analysed samples from 15 additional locations (Suppl. material 3). Specimens were morphologically examined, and at least one individual per sample was sequenced, as described in the subsequent sections.

### Morphometric analyses

The specimens were partly dissected in glycerol, and mounted on slides in glycerol gelatine. The animals were observed under a stereomicroscope Olympus SZX9 and a light microscope Zeiss Primo Star. For measurements, photographs and measurements were made using the program cellSense (Olympus); details on landmarks and overview of taxonomic characters are presented in Fišer et al. (2009c). Illustrations were prepared following digital inking (Coleman 2003, 2009) in Adobe Illustrator CS6, using photos as background pictures (taken on a Leica M205C with a mounted Canon EOS 5D Mark III).

### Molecular and phylogenetic analysis

Genomic DNA was isolated from one of the pereiopods or the whole animal (depending on specimen size) using the GenElute Mammalian Genomic DNA (Sigma-Aldrich, United States). We amplified the mitochondrial cytochrome oxidase I (COI) gene and three nuclear DNA gene fragments: part of 28S rRNA gene (28S), histone H3 (H3) and internal transcribed spacer I and II (ITS). A 660 bp long fragment of COI was amplified using primers LCO 1490 and HCO 2198 (Folmer et al. 1994); the part of 28S using primers from Verovnik et al. (2005) and Zakšek et al. (2007) and the H3 gene using primers from Colgan et al. (1998). PCR cycling conditions were the same as described in Fišer et al. (2013). For one of the focal species in this study, a subset of samples was selected for which about 2100 bp long fragments of the complete ITS region, including the flanking proportions of the 18S and 28S genes, were amplified using primers and procedures described by Flot et al. (2010). PCR products were purified using Exonuclease I and FastAP (Thermo Fisher Scientific Inc., United States) according to the manufacturer’s instructions. Each fragment was sequenced in both directions using PCR amplifications primers by Macrogen Europe (Amsterdam, Netherlands). An exception was the ITS fragment, which was sequenced using two additional primer pairs: i) ITS sf1– ITS sr1 and ii) ITS sf2– ITS sr2 (Flot et al. 2010). Chromatograms were assembled and edited using Geneious 11.0.3 (Biomatters, New Zealand).

### Phylogenetic analyses

The data for three gene fragments (COI, 28S and H3) were complemented with available sequences from previous studies, with the aim of including different phylogenetic lineages and potentially closely related taxa (Altermatt et al. 2014, Fišer et al. 2017). The dataset for phylogenetic analysis included 126 specimens and 98 taxa (Suppl. material 1). *Microniphargus leruthi* Schellenberg, 1934, the nearest taxon to the genus *Niphargus*, was used as outgroup. Sequences of all sequenced loci were aligned using MAFFT v.7 (Katoh and Standley 2013). The total length of the concatenated dataset was 1953 bp. We searched for the best-fitting substitution models and partitioning scheme using PartitionFinder 2.1.1 (Lanfear et al. 2012). Phylogenetic relationships were reconstructed using Bayesian inference with partition-specific settings in MrBayes 3.2.6 (Ronquist and Huelsenbeck 2003). A Bayesian MCMC tree search with two independent runs with four chains for each was run for 20 million generations, trees were sampled every 1000 generations. After reaching the stationary phase, the first 25% of trees were discarded, and from the remaining trees a 50% majority rule consensus tree was calculated. Phylogenetic analyses were run on the CIPRES Science Gateway (Miller et al. 2010, accessible at www.phylo.org).

### Species delimitation procedures

The selection of species delimitation methods critically depends on the species concept used. We applied the general lineage species concept, which states that species emerge as independently evolving segments of metapopulations (de Queiroz 2007). Within this concept, a species-taxon is delimited on evidence for a lack of gene flow among segments of the metapopulation. As such, it provides a broad testable framework for different spatial and ecological contexts of speciation, using different lines of evidence (e.g., characters, for more details see discussion in Fišer et al. 2018). The concept has been successfully and broadly used in *Niphargus* taxonomy (Fišer et al. 2009b).

Two different molecular-based species delimitation methods were applied: a distance-based Automatic Barcode Gap Discovery (ABGD) (Puillandre et al. 2012) and a tree-based Poisson Tree Processes (PTP) and Bayesian PTP (bPTP) model (Zhang et al. 2013). ABGD is an automated procedure that clusters sequences into candidate species based on pairwise distances by detecting differences between intra- and interspecific variation (i.e., barcoding gap) without *a priori* species hypothesis. ABGD analyses were performed at the ABGD web-server (http://wwwabi.snv.jussieu.fr/public/abgd/abgdweb.html) and analysed for COI sequences using the two available models Jukes-Cantor (JC69) and the Kimura K80, and three different values of relative gap width (X = 1.5, 1, 0.5). For the PTP and bPTP analyses we generated a new dataset from COI sequences used in this study combined with the largest available COI dataset for *Niphargus* (Eme et al. 2018). The maximum likelihood phylogenetic tree was generated using 855 haplotypes in PhyML 3.0 available at http://www.atgc-montpellier.fr/phyml/ (Guindon et al. 2010) using the GTR+I+G evolutionary model. We used the resulting phylogenetic tree as input for PTP and bPTP analyses. Calculations were conducted on the PTP webserver (http://species.h-its.org/ptp/) , with 500,000 MCMC generations, thinning set to 100 and burn-in at 25% and performing a Bayesian search.

In one focal species clade, an additional nuclear marker (ITS) was applied which provides a higher level of genetic variation and combined with mitochondrial COI (Suppl. material 2). The presence of species level lineages in sequence variation within the *N. tonywhitteni* sp. n. – *N. thienemanni* clade was also assessed by means of statistical parsimony (Templeton et al. 1992). Haplotype networks were built for both COI and ITS sequences using PopART software at http://popart.otago.ac.nz (Leigh and Bryant 2015).

The molecular delimitations were revised within the respective spatial context and with analyses of morphological variation. We searched for ecological differences in localities, and diagnostic morphological traits. Considering the paucity of the data, we could not apply statistical tests on the latter.

### Identification tools

In order to ease future molecular species identification, we revised the molecular data for *Niphargus* species reported from Switzerland. For species with unambiguous taxonomy we submitted their COI sequences to the Barcode of Life Data System (BOLD). Some species complexes await taxonomic evaluations. For these, a list of 28S sequences available at GenBank was compiled such that potential taxonomy-users can at least approximately identify the respective lineages.

In order to ease future analyses of morphological variation, we constructed a database in DELTA (Dallwitz et al. 1999). DELTA allows morphological characterization of species with both quantitative and qualitative morphological characters, and this information can be easily converted into species descriptions and dichotomous or interactive identification keys (Coleman et al. 2010).

## Results and discussion

### Faunistics

**Figure 1.**
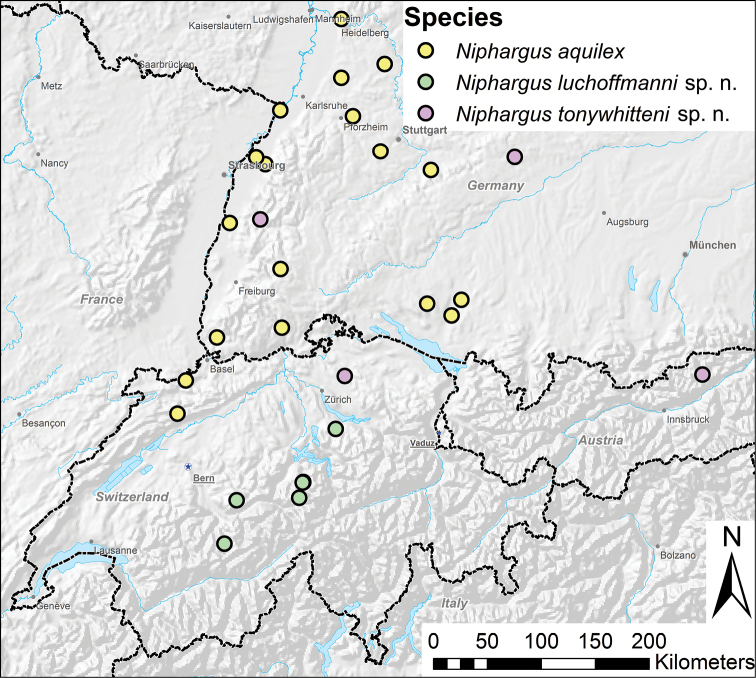
Finding sites of the new species *N. tonywhitteni* sp. n. (purple) and *N. luchoffmanni* sp. n. (green), and the distribution of *N. aquilex* (yellow) in Southern Germany and Northern Switzerland. Data source: Esri, 2013: Data & Maps for ArcGIS for use with Esri software; Elevation map of Europe EEA, Copenhagen, 2004.

New samples yielded mostly species reported from previous studies: *N. styx* Fišer, Konec, Alther, Švara & Altermatt, 2017, *N. puteanus* (Koch, 1836), *N. thienemanni* Schellenberg, 1934, *N. rhenorhodanensis* Schellenberg, 1937, a species labelled as N. cf. thienemanni in Fišer et al. (2017), and *N. luchoffmanni* sp. n. (see section “Species descriptions” below) labelled as N. cf. stygius 1 in Fišer et al. (2017) (see Suppl. material 3). In addition, the new samples unveiled a species that morphologically corresponds to the description of *N. aquilex* Schiødte, 1855. We could not verify its molecular identity, however, this is the first finding and confirmation of this species in Switzerland after decades (Strinati 1966). Interestingly, the new finding is not far from the first finding place (Fig. 1). The number of *Niphargus* species from Switzerland has risen to 22. Still unresolved is the status of two taxa, likely distinct and undescribed species, so far only represented by two and one individual, and provisionally labelled as N. cf. thienemanni and N. cf. stygius, respectively. Another problem is the paraphyletic complex *N. rhenorhodanensis*, with at least five distinct lineages, many of which may comprise more than one species, and their occurrence in Switzerland is not yet resolved (Table 1).

**Table 1.** A check list of *Niphargus* species from Switzerland, with an overview of the diagnostic traits.

List of *Niphargus* species of Switzerland	Reference sequence (28S) for lineage identifcation GenBank Access. No. ^1^	Reference sequence (COI) for species identifcation GenBank Access. No. ^1^	Morphological information available in DELTA ^2^
*Niphargus aquilex* Schiødte, 1855	/	/	yes
*Niphargus auerbachi* Schellenberg, 1934	EU693292	KX379130	yes
*Niphargus brixianus* Ruffo, 1937	KX379011	KX379109	yes
*Niphargus caspary* Pratz, 1866	KX379003	KX379123	yes
**^3^** Niphargus cf. stygius	KX379016	KX379103	yes
**^3^** N. cf. thienemanni Schellenberg, 1934	KX379031	KX379074	yes
*Niphargus forelii* Humbert, 1877	/	/	yes
*Niphargus inopinatus* Schellenberg, 1932	/	KY707004	yes
*Niphargus luchoffmanni* sp. n.	KX379014	KX379105	yes
*Niphargus muotae* Fišer, Konec, Alther, Švara & Altermatt, 2017	KX379024	KX379095	yes
*Niphargus murimali* Fišer, Konec, Alther, Švara & Altermatt, 2017	KX379022	KX379097	yes
*Niphargus puteanus* Koch, 1836	MH172402	MH172434	yes
**^3^** *Niphargus rhenorhodanensis* complex Schellenberg, 1937, lineage ABC	KJ566681	KX379117	On a level of complex
**^3^** *Niphargus rhenorhodanensis* complex Schellenberg, 1937, lineage FG	KX379042	KX379084	On a level of complex
*Niphargus rhenorhodanensis complex Schellenberg, 1937, lineage H	KJ566685	KX379116	On a level of complex
**^3^** *Niphargus rhenorhodanensis* complex Schellenberg, 1937, lineage JK	MH172416	MH172436	On a level of complex
*Niphargus setiferus* Schellenberg, 1937	/	/	yes
*Niphargus styx* Fišer, Konec, Alther, Švara & Altermatt, 2017	KX379023	KX379096	yes
*Niphargus thienemanni* Schellenberg, 1934	KJ566688	KX379114	yes
*Niphargus thuringius* Schellenberg, 1934	/	KY706717	yes
*Niphargus tonywhitteni* sp. n.	KX379045	KX379081	yes
*Niphargus virei* B. Chevreux, 1896	KJ566680	KX379098	yes

**^1^**The diagnostic traits of two genes; accessible via GenBank and BOLD. **^2^**Morphological diagnostic traits are available in DELTA database. **^3^**The taxonomy of the species is not resolved yet; their identity of the species can be assessed only to lineage level.

### Molecular and phylogenetic analysis, species delimitation, and barcodes

**Figure 2.**
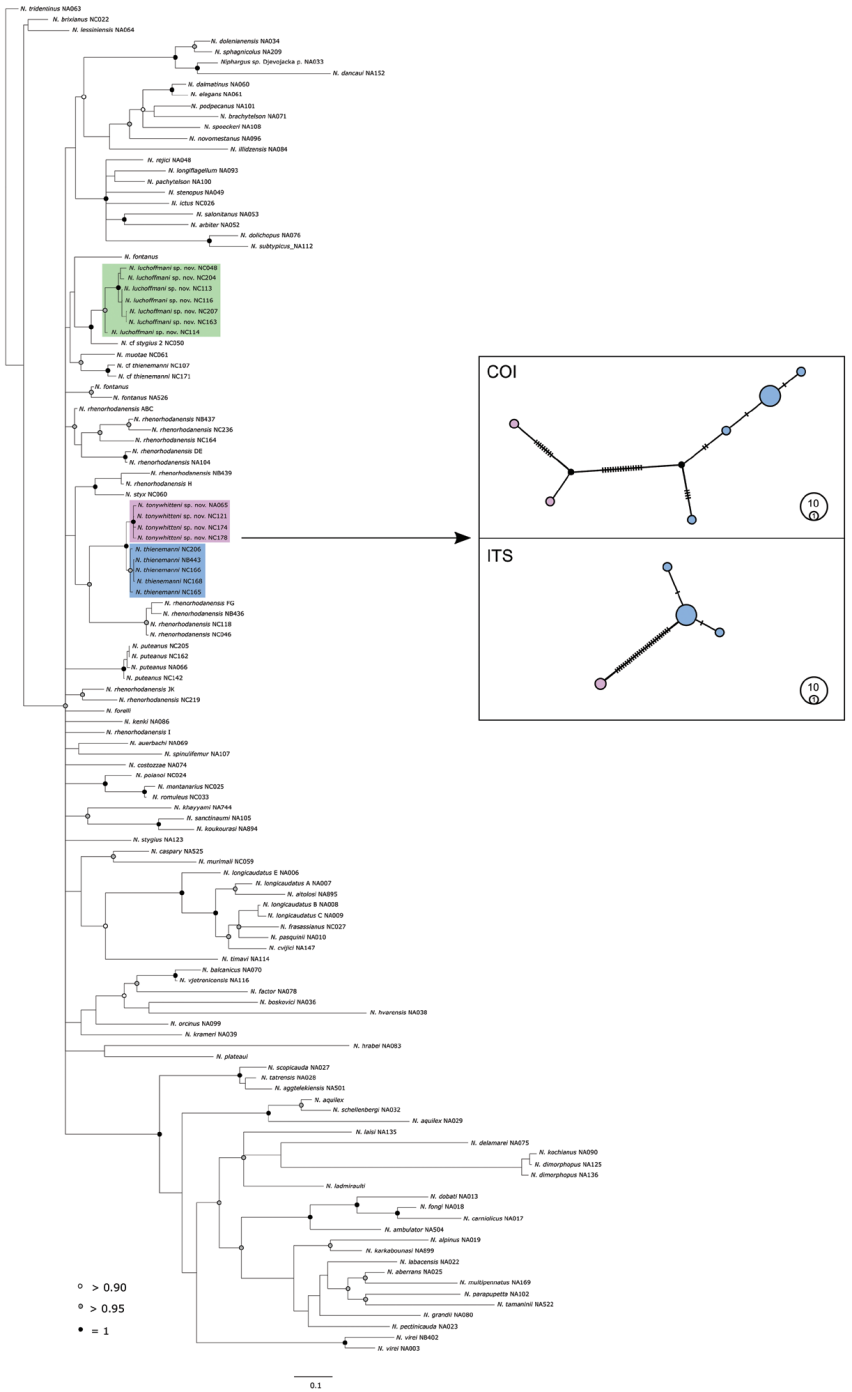
Phylogenetic relationships of *Niphargus* species focusing on new taxa from Switzerland. Highlighted are two new species and a sister species (green: *N. luchoffmanni* sp. n., blue: *N. thienemanni*, purple: *N. tonywhitteni* sp. n.) The tree was constructed using Bayesian inference on COI, 28S rRNA and histone gene sequences. The tree was rooted using *Microniphargus leruthi* (not presented). On the right side, two haplotype networks (based on COI and ITS) calculated within *N. thienemanni* and *N. tonywhitteni* sp. n. species pairs are shown. These networks are based on a higher number of samples (N=10, see Suppl. material 2) compared to the phylogenetic tree. Colours in the networks correspond to colours in the tree.

Phylogenetic analyses included new samples of *Niphargus* from Switzerland (12 additional individuals; sequences from three samples could not be obtained). The additional samples did not affect phylogenetic structure (Fig. 2) and the newly obtained phylogeny showed no substantial differences from the previously published one (Fišer et al. 2017). The phylogenetic position of the two herein described species, however, deserves more discussion.

The first species, *Niphargus tonywhitteni* sp. n. (see section “Species description” below), is closely related to *N. thienemanni* (Fig. 2, blue and purple shading). Genetic differences between the two sister species are relatively small, but distinct. ABGD analysis using default value of relative gap width (1.5) suggested that the lineage comprised a single species. By contrast, lowering the threshold to 0.5 (also used in Fišer et al. 2017) suggested that the lineage was comprised of two species. This result was concordant with results from the PTP and bPTP analyses. The results of PTP and bPTP did not differ from each other and both analyses identified two species within this lineage. A more detailed network analysis reinforced the hypothesis that the lineage is comprised of two species. The patterns in differentiation of mitochondrial COI and molecular ITS markers were congruent, and both networks suggest there is no indication of gene flow between the two species (Fig. 2). In addition, the analysis of field notes implied that the two species might differ ecologically (Fig. 1). *Niphargus thienemanni* was found exclusively in springs, above 1395 m a. s. l. By contrast, the hitherto undescribed putative species lived in interstitial habitats, linked to alluvial plains of the Rhine and the Danube. The two species also differed morphologically (details in the following section). We therefore concluded that *N. thienemanni* and *N. tonywhitteni* sp. n. needed to be treated as two distinct, albeit only recently evolved species. The subtle morphological differences, and small genetic distances imply that the two species split relatively recently, perhaps when post-Pleistocene warming and glacier melting made previously non-occupied habitats on higher elevations accessible for colonization.

The second species, *N. luchoffmanni* sp. n. (see section “Species description” below), belongs to a lineage endemic to Switzerland that comprises two sister species, namely *N. luchoffmanni* sp. n. and an as yet undescribed species provisionally named N. cf. stygius (an insufficient number of specimens for the latter taxon does not allow a proper description yet). The results of species delimitation analyses (ABGD and PTP) of COI approved their separate species status. We could not assess morphological differences between the two, nor analyse their detailed genetic differentiation, as we had only one damaged male of N. cf. stygius. Yet, genetic distinctness (0.045 K2P) suggested that the two species differed to such an extent that interbreeding between them is unlikely (Lagrue et al. 2014). A substantial within-species genetic variation was found, but this was still significantly lower compared to distance to N. cf. stygius.

All available COI sequences of *Niphargus* species from Switzerland were submitted to GenBank and can be accessed also through BOLD. Their accession numbers are MH172382-MH 172398 and MH172401-MH172436 and can be viewed in Table 1.

### Species descriptions

#### Niphargus tonywhitteni sp. n.

Taxon classificationAnimaliaAmphipodaNiphargidae

http://zoobank.org/E5CE0D3A-2BE9-4794-851F-D1D537EEE767

**Figure 3.**
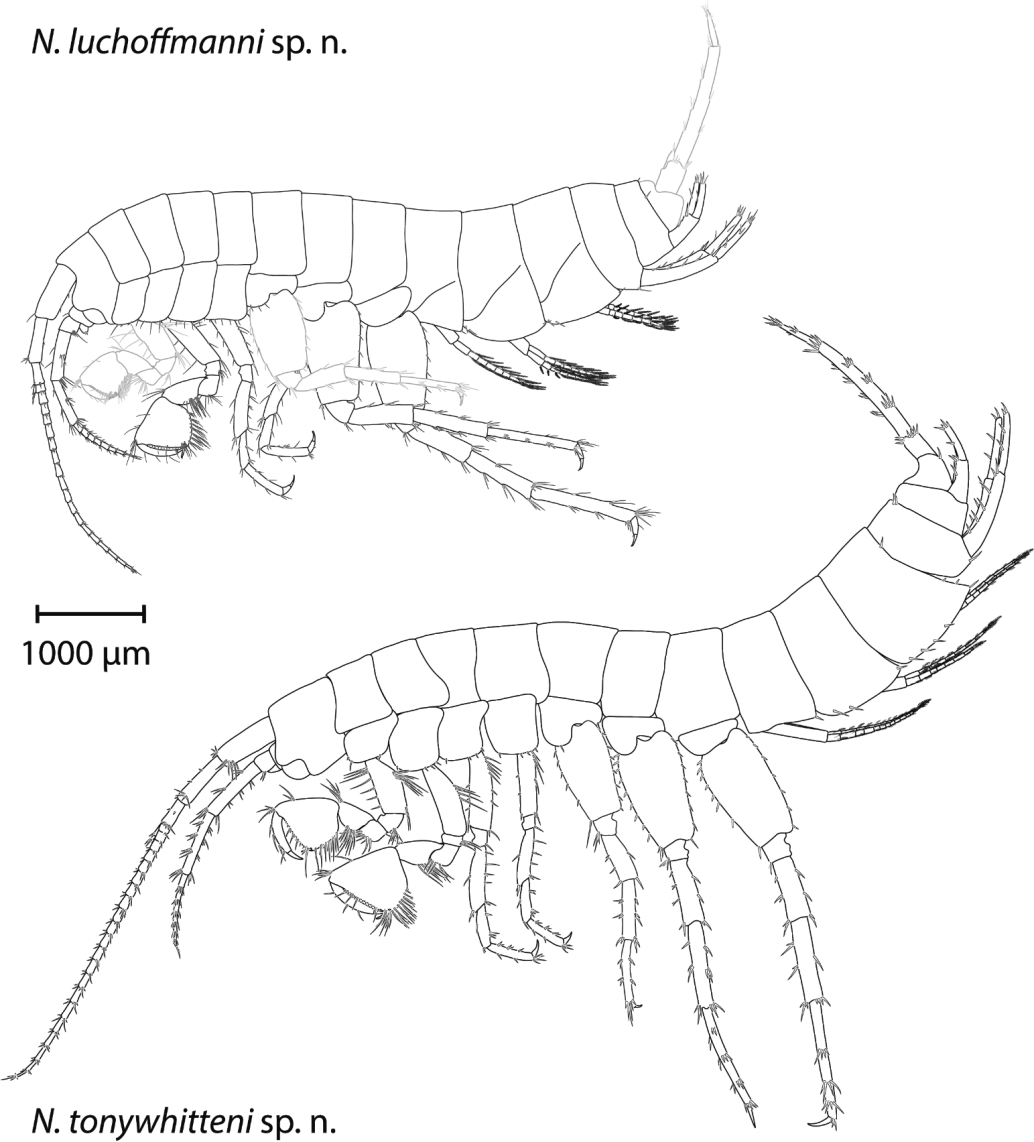
Two new *Niphargus* species from Switzerland. The drawings are scaled to the same size. Top: *N. luchoffmanni* sp. n. (holotype, male 6.7 mm), bottom: *N. tonywhitteni* sp. n. (holotype, male 9.1 mm). Both specimens were rearranged digitally after drawing. Missing parts were taken from the right hand side of the specimen and are depicted in grey.

**Figure 4.**
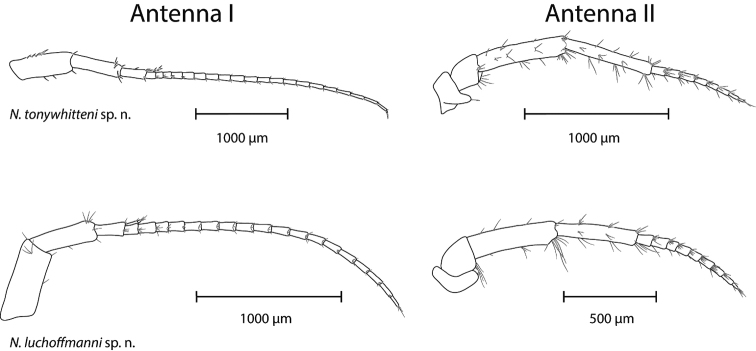
Antenna I (left) and II (right) of *N. tonywhitteni* sp. n. (top, holotype, male 9.1 mm) and *N. luchoffmanni* sp. n. (bottom, holotype, male 6.7 mm). Drawings are not scaled to the same size.

**Figure 5.**
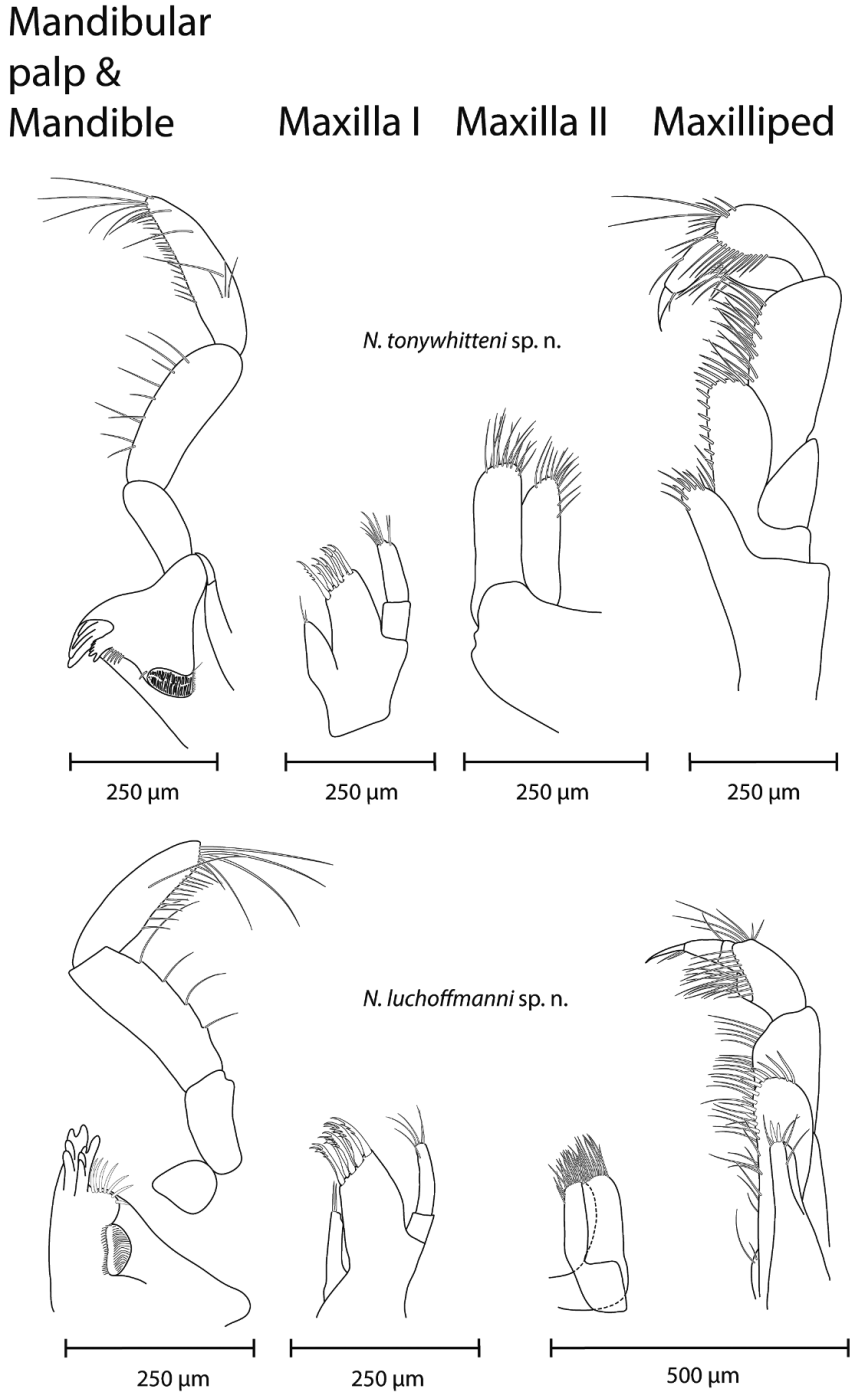
Mouth parts (mandible & mandibular palp, maxilla I, maxilla II, maxilliped; from left to right) of *N. tonywhitteni* sp. n. (top, holotype, male 9.1 mm) and *N. luchoffmanni* sp. n. (bottom, holotype, male 6.7 mm). Drawings are not scaled to the same size.

**Figure 6.**
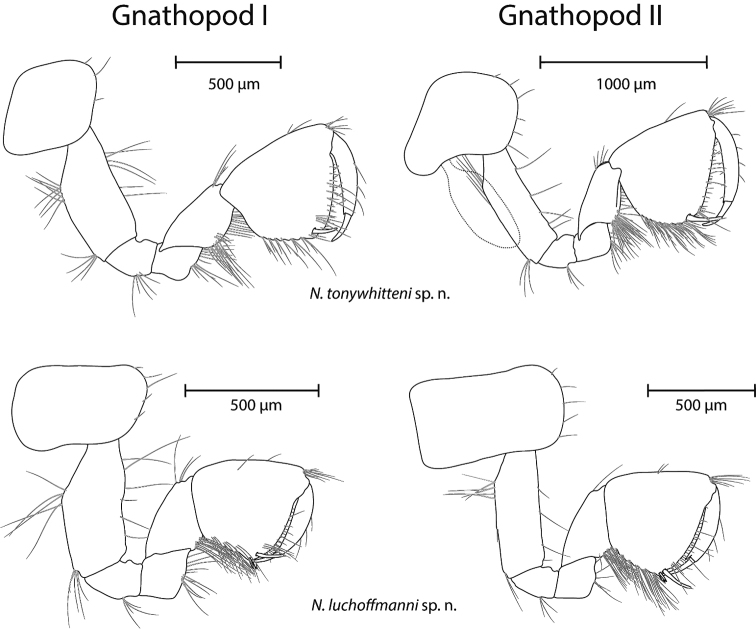
Gnathopod I (left) and II (right) of *N. tonywhitteni* sp. n. (top, holotype, male 9.1 mm) and *N. luchoffmanni* sp. n. (bottom, holotype, male 6.7 mm). Gills are dashed, and drawn only when intact (missing in *N. luchoffmanni* sp. n.). Drawings are not scaled to the same size.

**Figure 7.**
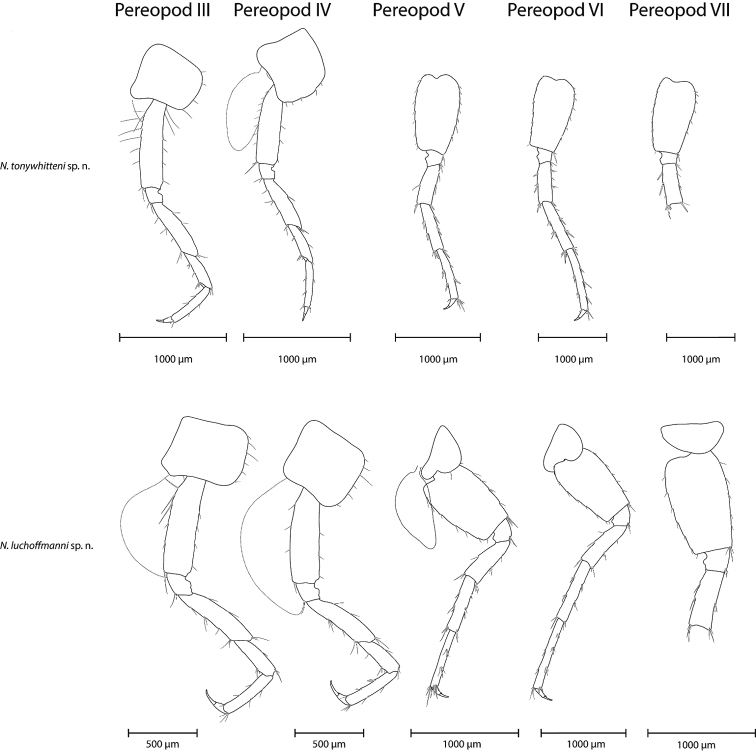
Pereopods III-VII (from left to right) of *N. tonywhitteni* sp. n. (top, holotype, male 9.1 mm) and *N. luchoffmanni* sp. n. (bottom, holotype, male 6.7 mm). Pereopods VII on this side were broken; see Fig. 3 for illustration of the entire pereopod. Gills were only drawn when intact; however, they are present on pereopods III-VI. Drawings are not scaled to the same size.

**Figure 8.**
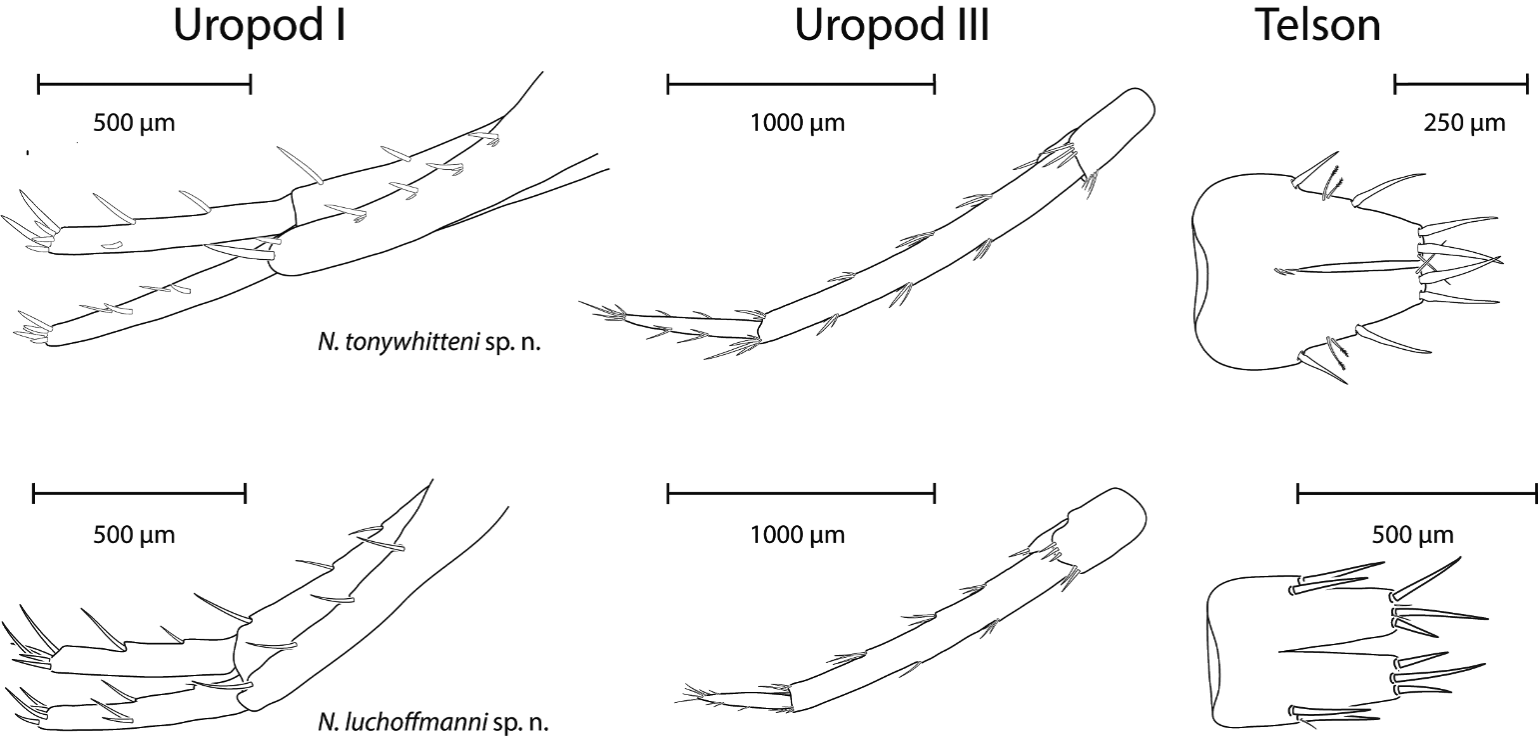
Uropod I (left) and III (middle), and Telson (right) of *N. tonywhitteni* sp. n. (top, holotype, male 9.1 mm) and *N. luchoffmanni* sp. n. (bottom, holotype, male 6.7 mm). Drawings are not scaled to the same size.

Figs 3 , 4 , 5 , 6 , 7 , 8

##### Holotype.

Male, 9.1 mm. The specimen is mounted on two slides and deposited in the collection of the Musée de Zoologie, Lausanne, Switzerland under voucher number GBIFCH00585714 and GBIFCH00585715. Sampled on 17 October 2014 by Tom Gonser. Paratypes represent one male of length 7.5 mm with voucher numbers GBIFCH00587517.

##### Material examined.

Three males of lengths 9.1, 7.5 and 9.1 mm; specimens are partially dissected and mounted on slides with voucher numbers GBIFCH00585714, GBIFCH00585715, and GBIFCH00587517; three other specimens were sequenced.

##### Type locality.

Gravel bed of Töss River near Winterthur, Switzerland (CH1903: 697,715/257,410)

##### Diagnosis.

Small *Niphargus*, of mid-slender appearance closely resembling *N. fontanus*. Telson narrow, with long apical and lateral spines; dorsal spines lacking. Propodus of gnathopod I of rectangular shape, propodus of gnathopod II almond (hoof) shape. Uropods I with equal rami; uropod III rod shaped, likely sexually dimorphic, with elongated distal article.

##### Description

**(based on dissected specimens).** *Head and trunk* (Figs 3, 8). Body length up to 9.1 mm. Head length approximately 10% of body length; rostrum absent. Pereonites I–VI without setae, pereonite VII with one seta ventro-posteriorly.

Pleonites I–III with up to four setae along the entire respective dorso-posterior margins. Epimeral plate II only slightly inclined, posterior and ventral margins slightly sinusoid and convex, respectively; ventro-postero-distal corner distinct; two spines along ventral margin; four setae along posterior margin. Epimeral plate III inclined, posterior and ventral margin sinusoid and convex, respectively; ventro-postero-distal corner distinct but not produced; two spiniform setae along ventral margin; four thin setae along posterior margin.

Urosomite I postero-dorso-laterally with one strong spiniform seta sometimes accompanied with one slender and flexible seta; urosomite II postero-dorso-laterally with two to three strong spiniform setae; urosomite III without setae. At the base of uropod I a single strong spiniform seta.

Telson length : width ratio is 1 : 0.85–0.90; cleft is 0.6–0.65 telson length; telson margins straight and narrowing apically. Telson spiniform setae (per lobe, left-right lobe asymmetry commonly observed): three to five apical, and none to two lateral spiniform setae; dorsal and mesial setae were not observed. Apical spiniform setae up to 0.5 telson length. Pairs of plumose setae laterally.

*Antennae* (Fig. 4). Antenna I 0.45–0.55 of body length. Flagellum with 21 articles; each article with one aesthetasc. Peduncle articles in ratio 1 : 0.85–0.90 : 0.41–0.45. Proximal article of peduncle dorso-distally slightly produced. Accessory flagellum biarticulated; distal article shorter than one quarter of proximal article length.

Length ratio antenna I : antenna II as 1 : 0.46–0.47. Flagellum of antenna II with seven to eight articles; each article with setae and elongated sensillae of unknown function. Peduncle articles lengths 4 : 5 is 1 : 0.93–0.98; flagellum 0.55-0.58 times length of peduncle articles 4+5.

*Mouthparts* (Fig. 5). Labrum typical; inner lobes of labium hardly visible.

Left mandible: incisor with five teeth, lacinia mobilis with four teeth; between lacinia and molar a row of serrated setae, few spatulate setae and one long seta at the base of molar. Right mandible: incisor processus with four teeth, lacinia mobilis with several small teeth, between lacinia and molar a row of thick serrated setae. Ratio of mandibular palp article 2 : article 3 (distal) is 1 : 1.12–1.22. Proximal palp article without setae; second article with seven to nine setae; distal article with a group of four A setae; three groups of B setae; 18–19 D setae and five E setae.

Maxilla I distal palp article with seven to eight apical setae. Outer lobe of maxilla I with a row of seven stout setae, inner with many subapical denticles, the remaining setae with one denticle; inner lobe with two apical setae.

Maxilla II inner lobe slightly smaller than outer lobe; both lobes setose apically.

Maxilliped palp article 2 with five to eight rows of setae along inner margin; distal article with a dorsal seta, and setae at the base of nail. Maxilliped outer lobe with seven to eight stout setae mesially to subapically, and three setae apically; inner lobe apically with two stout setae and six serrated setae.

*Coxal plates, and gills* (Figs 3, 6, 7). Coxal plate I of parallelogram shape, with rounded antero-ventral corner and armed with three to four setae. Coxal plates II–IV width : depth ratios are 1.09–1.16 : 1, 0.87–0.89 : 1 and 0.85–0.92 : 1 respectively; anterior and ventral margins with five to six, four and four to five setae respectively. Coxal plate IV posteriorly distinctly concave. Coxal plates V–VI anteriorly with large lobe; posterior margins with one seta. Coxal plate VII half-rounded shaped with one posterior seta. Gills II–VI ovoid.

*Gnathopod I* (Fig. 6). Ischium with one group of two to six postero-distal setae. Carpus 0.58–0.61 of basis length and 0.77–0.80 of propodus length; broadened distally. Carpus with single distal group of setae anteriorly; transverse rows of setae along posterior margin and a row of setae postero-laterally. Propodus rectangular. Along posterior margin five to six rows of setae. Anterior margin with two to three groups of total 11–12 setae in addition to antero-distal group of seven to eight setae. Several groups of short setae on the inner surface present. Palmar corner armed with a long spiniform palmar seta, three serrated spiniform setae, a single supporting spiniform seta on inner surface and three to four long setae below palmar spine. Palm setose. Nail length 0.31–0.32 of total dactylus length; four to six setae along anterior margin; a row of short setae along inner margin.

*Gnathopod II* (Fig. 6). Basis width : length is 0.31–0.32 : 1. Ischium with four postero-distal setae. Carpus 0.56–0.58 of basis length and 0.75–0.85 of propodus length, distally broadened. Carpus with distal group of setae anteriorly; few transverse rows of setae along posterior margin and a row of setae postero-laterally. Propodus of hoof or almond shape, large (circumference measures up to 0.19–0.20 of body length), larger than propodus of gnathopod I (I : II as 0.79–0.81 : 1). Posterior margin with eight to nine rows of setae. Anterior margin with a pair of individual setae in addition to eight to nine antero-distal setae. Individual surface setae present. Palmar corner with one strong palmar spiniform seta, single supporting spiniform seta on inner surface and one to two denticulated thick spiniform setae on outer side. Palm setose, below spiniform palmar seta a group of three long setae. Nail length 0.29–0.36 of total dactylus length; four setae along anterior margin; few short setae along inner margin.

*Pereopods III-IV* (Fig. 7). Lengths of pereopods III and IV subequal. Dactylus IV 0.34–0.43 of propodus IV; nail length 0.47–0.50 of total dactylus length. Dactyli III–IV with dorsal plumose seta; two tiny setae at the base of nail.

*Pereopods V–VII* (Fig. 7). Lengths of pereopods V : VI : VII is 1 : 1.30–1.33 : 1.30–1.41; pereopod VII measures 0.44–0.48 of body length.

Bases V–VII broad, respective length : width ratios as 1 : 0.60–0.65, 1 : 0.55–0.62 and 1 : 0.57–0.62; posterior margins straight to convex; bases V–VII with moderate large posterior lobes; posteriorly eight to nine, eight to ten and seven to nine setae, respectively; anteriorly seven to eight, eight and seven to eight groups of spines, respectively. Dactyli V–VII with dorsal plumose seta, with two tiny setae at the base of the nail.

*Pleopods and uropods* (Fig. 8). Pleopods I–III with two hooked retinacles. Pleopod II rami with seven to eight and nine to ten articles.

Uropod I protopodite with six dorso-lateral spiniform setae and three dorso-medial spiniform setae. Exopodite : endopodite lengths is 1 : 1.0–1.03; rami straight. Endopodite with three individual spiniform setae laterally and five spiniform setae apically. Exopodite with five groups of totally nine setae; mesially with individual spiniform setae and laterally with spiniform and flexible setae; five spiniform setae apically.

Uropod II exopodite : endopodite lengths is 1 : 1.09.

Uropod III rod-shaped, 0.25–0.30 of body length. Protopodite with none to one lateral setae and seven to nine apical spiniform setae. Endopodite 0.54–0.61 of protopodite length, laterally without setae, apically with two setae, at least one spiniform. Exopodite of uropod III distal article 0.35–0.41 of the proximal article length. Proximal article with four to six groups of thin-flexible, spiniform and plumose setae along inner margin and four to five groups of thin-flexible and spiniform setae along outer margin. Distal article with two to three groups of thin-flexible setae along each margin, and a pair of setae apically.

##### Etymology.

The species is named in honour of Tony Whitten (1953–2017), who devoted his life to nature conservation including conserving life in caves. He was a co-chair of the Cave Invertebrate Specialist Group at IUCN.

##### Habitat and distribution.

The species is known only from interstitial or related habitats. The species was found along the northern margin of the Alpine arch, between Achensee in Austria, Southern Germany and the type locality in Switzerland.

##### Variability.

Only a small sample was available, not all individuals were adult, and many specimens were damaged. The extent of sexual dimorphism in uropod III is unknown; the terminal article of exopodite indicates elongation, as in *N. fontanus* from Great Britain, and our observations suggest that this article is longer in males and shorter in females. Most variation noticed can be likely attributed to different sizes of the specimens.

##### Remarks and affiliation.

The species is closely related to *N. thienemanni*, from which it clearly differs by the almond-hoof shape of propodus of gnathopod II (rectangular in *N. thienemanni*). However, the newly described species is strikingly similar to *N. fontanus* Bate, 1859 from Great Britain, Belgium and France. The latter comprises a complex of cryptic species, distributed between Great Britain and Alps (McInerney et al. 2014), whereas the newly described *N. tonywhitteni* sp. n. belongs to a completely different phylogenetic lineage (Fig. 2), ruling out a possible conspecificity. The morphological differences between the two complexes are difficult to evaluate, mainly because we have only limited insights into variation of *N. tonywhitteni* sp. n. as well as the species complex containing the nominal species. We compared the newly described species with the lectotype and information available in various descriptions (Ginet 1996, Hartke et al. 2011). The only observed difference is in the shape of propodus of gnathopod I, which tends to be more rectangular in the newly described species in contrast to more almond shaped propodus of the nominal lineage. Additional identification traits depend on non-morphological information, i.e., geographic origin of the species, and especially on diagnostic COI sequences. While the description of *N. tonywhitteni* sp. n. substantially improved the knowledge of *Niphargus* in Switzerland, it is clear that the polyphyletic complex *N. fontanus – N. tonywhitteni* sp. n. is awaiting revision, which is beyond the scope of the present paper.

#### Niphargus luchoffmanni sp. n.

Taxon classificationAnimaliaAmphipodaNiphargidae

http://zoobank.org/E1C7C812-1494-40A8-8844-C6DC45C7AF07

Figs 3 , 4 , 5 , 6 , 7 , 8

##### Holotype.

Male, 6.7 mm. The specimen is mounted on two slides and deposited in the collection of the the Musée de Zoologie, Lausanne, Switzerland under voucher numbers GBIFCH00585716 and GBIFCH00585717. Sampled on May 29, 2014 by Verena Lubini. Additional paratypes include 9.15 mm long and partially dissected female deposited under voucher number GBIFCH00587519, a male 6.8 mm long deposited under voucher number GBIFCH00587518 and several un-dissected specimens deposited in vials under GBIFCH00329353 and GBIFCH00329354.

##### Material examined.

Two males of lengths 6.7 and 6.8 mm and a female 9.15 mm long; specimens are partially dissected and mounted on slides with voucher numbers GBIFCH00585716, GBIFCH00585717, GBIFCH00587518 and GBIFCH00587519; seven other specimens were sequenced.

##### Type locality.

Marchbachquelle, Wolfenschiessen, Switzerland (CH1903: 672,490/190,300).

##### Diagnosis.

Mid-sized species, in general appearance similar to *N. forelii*. Epimeral plates angular. Telson with three long apical spines, one lateral, and one dorsal spine per lobe. Propods of gnathopods I and II of rectangular shape, propodus of gnathopod II large when compared to body length and propodus I. Maxilla outer lobe with seven spiniform setae, the inner four comb-like with long subapical denticles, the remaining three spines with one such denticle. Uropod I inner ramus slightly shorter than outer ramus; uropod II inner ramus slightly longer than outer ramus. Uropod III distal article elongated in males, as long as 0.5 times proximal article.

##### Description

**(based on dissected specimens).** *Head and trunk* (Fig. 3). Body length up to 9.2 mm. Head length approximately 10% of body length; rostrum absent. Pereonites I–VI without setae, pereonite VII with one seta ventro-posteriorly.

Pleonites I–III with up to four setae along the entire respective dorso-posterior margins. Epimeral plate II only slightly inclined, posterior and ventral margins slightly convex; ventro-postero-distal corner distinct; two spines along ventral margin; three to six setae along posterior margin. Epimeral plate III inclined, posterior and ventral margin concave and convex, respectively; ventro-postero-distal corner distinct but not produced; two to three spiniform setae along ventral margin; four to five thin setae along posterior margin.

Urosomite I postero-dorso-laterally with one slender and flexible seta; urosomite II postero-dorso-laterally with one strong spiniform setae accompanied with one slender and flexible seta; urosomite III without setae. At the base of uropod I, a single strong spiniform seta.

Telson length : width ratio is 1 : 0.81–0.91; cleft is 0.69–0.72 telson length; telson margins straight and narrowing apically. Telson spiniform setae (per lobe, left-right lobe asymmetry commonly observed): three apical, one dorsal and one lateral spiniform; mesial setae were not observed. Apical spiniform setae 0.44–0.5 telson length. Pairs of plumose setae laterally.

*Antennae* (Fig. 4). Antenna I 0.41–0.56 times body length. Flagellum with 17–20 articles; each article with one aesthetasc. Peduncle articles in ratio 1 : 0.79–0.87 : 0.37–0.47. Proximal article of peduncle dorso-distally slightly produced. Accessory flagellum biarticulated; distal article shorter than one quarter of proximal article length.

Length ratio antenna I : antenna II is 1 : 0.48–0.57. Flagellum of antenna II with nine to ten articles; each article with setae and elongated sensillae of unknown function. Peduncle articles lengths 4 : 5 is 1 : 0.93–0.95; flagellum 0.69–0.77 of length of peduncle articles 4 and 5.

*Mouthparts* (Fig. 5). Labrum typical; inner lobes of labium hardly visible.

Left mandible: incisor with five teeth, lacinia mobilis with four teeth; between lacinia and molar a row of serrated setae, few spatulate setae and a long seta at the base of molar. Right mandible: incisor processus with four teeth, lacinia mobilis with several small teeth, between lacinia and molar a row of thick serrated setae. Ratio of mandibular palp article 2 : article 3 (distal) is 1 : 1.01–1.11. Proximal palp article without setae; the second article with seven to eleven setae; distal article with a group of two to four A setae; two to three groups of B setae; 15–20 D setae and three E setae.

Maxilla I distal palp article with five to six apical setae. Outer lobe of maxilla I with a row of seven stout setae, inner four comb-like, with many long subapical denticles, the remaining three setae with one denticle; inner lobe with two to three apical setae.

Maxilla II inner lobe slightly smaller than outer lobe; both lobes setose apically.

Maxilliped palp article 2 with seven to eight rows of setae along inner margin; distal article with a dorsal seta, and setae at the base of nail. Maxilliped outer lobe with nine to eleven stout setae mesially to subapically, and three to five setae apically; inner lobe apically with three to four stout setae and seven serrated setae.

*Coxal plates, and gills* (Figs 3, 6, 7). Coxal plate I of parallelogram shape, with rounded antero-ventral corner and armed with four to six setae. Coxal plates II-IV width : depth ratios as 0.87–1.07 : 1, 1.03–1.12 : 1 and 0.96–1.13 : 1; anterior and ventral margins with four to seven, five to six and five to seven setae. Coxal plate IV posteriorly distinctly concave. Coxal plates V–VI anteriorly with large lobes; posterior margins with one seta. Coxal plate VII half-rounded shaped with one posterior seta. Gills II–VI ovoid.

*Gnathopod I* (Fig. 6). Ischium with one group of four to six postero-distal setae. Carpus 0.58–0.61 of basis length and 0.84–0.97 of propodus length; broadened distally. Carpus with single distal group of setae anteriorly, rarely accompanied by an additional seta in the mid of article; transverse rows of setae along posterior margin and a row of setae postero-laterally. Propodus rectangular. Along posterior margin, three to five rows of setae. Anterior margin with two to three groups of total four to eleven setae in addition to antero-distal group of eight setae. Several groups of short setae on the inner surface present. Palmar corner armed with a long spiniform palmar seta, two to three serrated spiniform seta, a single supporting spiniform seta on inner surface and three to five long setae below palmar spine. Palm setose. Nail length 0.31–0.32 of total dactylus length; four setae along anterior margin; a row of short setae along inner margin.

*Gnathopod II* (Figs 6). Basis width : length is 0.28–0.30 : 1. Ischium with three to four postero-distal setae. Carpus 0.52–0.57 of basis length and 0.83–0.91 of propodus length, distally broadened. Carpus with distal group of setae anteriorly, rarely accompanied by an additional seta in the middle of the article; few transverse rows of setae along posterior margin and a row of setae postero-laterally. Propodus rectangular, large (circumference measures up to 0.20–0.23 of body length), much larger than propodus of gnathopod I (I : II as 0.75–0.76 : 1). Posterior margin with six to eight rows of setae. Anterior margin with two to three groups of total four to six setae in addition to seven to ten antero-distal setae. Individual surface setae present. Palmar corner with one strong palmar spiniform seta, a single supporting spiniform seta on inner surface and two denticulated thick-spiniform setae on outer side. Palm setose, below spiniform palmar seta, a group of three to four long setae. Nail length 0.30–0.32 of total dactylus length; three to six setae along anterior margin; a few short setae along inner margin.

*Pereopods III–IV* (Fig. 7): Lengths of pereopods III and IV subequal. Dactylus IV 0.46–0.52 of propodus IV; nail length 0.52–0.59 of total dactylus length. Dactyli III-IV with a dorsal plumose seta; one spiniform seta at the base of nail, sometimes accompanied by a tiny seta.

*Pereopods V–VII* (Fig. 7): Lengths of pereopods V : VI : VII is 1 : 1.33–1.34 : 1.39–1.42; pereopod VII measures 0.55–0.59 of body length.

Bases V-VII broad, respective length : width ratios as 1 : 0.64–0.67, 1 : 0.60–0.65 and 1 : 0.60–0.63; posterior margins straight to convex; bases V-VII with moderate posterior lobes; posteriorly eight to eleven, nine to twelve and seven to ten setae, respectively; anteriorly six to seven, six and five to seven groups of spines, respectively. Dactyli V–VII with dorsal plumose seta; spiniform seta at the base of nail, in most cases accompanied by one tiny seta.

*Pleopods and uropods* (Fig. 8): Pleopods I–III with two hooked retinacles. Pleopod II rami with seven to nine and nine to ten articles.

Uropod I protopodite with three to six dorso-lateral spiniform setae and three to four dorso-medial spiniform setae. Exopodite : endopodite lengths is 1 : 0.82–0.99; rami straight. Endopodite with two individual spiniform setae laterally and five spiniform setae apically. Exopodite with two to four groups totalling three to eight setae; mesial groups comprise individual spiniform setae, whereas lateral groups comprise spiniform and flexible setae groups; apically five spiniform setae.

Uropod II exopodite : endopodite lengths is 1 : 1.02–1.12.

Uropod III rod-shaped, 0.22–0.41 of body length. Protopodite with one to two lateral setae and six to seven apical spiniform setae. Endopodite 0.45–0.50 of protopodite length, laterally with 0–1 seta, apically with two setae, at least one spiniform. Exopodite of uropod III distal article 0.28–0.48 of the proximal article length. Proximal article with four to six groups of thin-flexible, spiniform and plumose setae along inner margin and four groups of thin-flexible and spiniform setae along outer margin. Distal article with one to four groups of thin-flexible setae along each margin, and five to six of setae apically.

##### Etymology.

The species is named in honour of Hans Lukas “Luc” Hoffmann (1923–2016), naturalist and ecologists, who importantly influenced nature conservation worldwide. Among others, he was the founder of the MAVA foundation and co-founder of the World Wide Fund for Nature (WWF).

##### Habitat and distribution.

The species has been hitherto reported from springs, and seems to be endemic to central Switzerland (Fig. 1).

##### Variability.

The variability of the species is poorly understood, as we could analyse relatively little material, with numerous sub-adult and damaged specimens. Males and females differ in length of distal article of uropod III, which is remarkably longer in males. Larger specimens tend to have narrower bases of pereopods V–VII. The pattern of denticulation on spines on outer lobe of maxilla I is, however, stable and the most important diagnostic trait.

##### Remarks and affiliation.

In a morphological sense, *N. luchoffmanni* sp. n. shows some similarities to *N. forelii* Humbert, 1876. We compared *N. luchoffmanni* sp. n. with neotypes from Bodensee from Berlin Museum and species descriptions (Karaman and Ruffo 1993, Ginet 1996). Both species have long dactyls, long telson spines, multiple setae along gnathopod dactyls, an elongated distal article of uropod III in males, and the endopodite of uropod I shorter than the exopodite. Yet, there are few distinct traits separating both species. The differences in sizes of propods of gnathopods I and II is more pronounced in *N. luchoffmanni* sp. n. than in *N. forelii*. In addition, endopodite of the uropod II is longer and shorter than the exopodite in *N. luchoffmanni* sp. n. and *N. forelii*, respectively. *Niphargus luchoffmanni* sp. n. has one dorsal spine on telson, while *N. forelii* is lacking dorsal telson spines. Finally, the spines on the outer lobe of maxilla I are different: while *N. luchoffmanni* sp. n. has at least four spines multidenticulate, *N. forelii* has, at most, one such spine.

### DELTA database

The present database counts 19 out of 22 species and 19 characters treated for each species. In the database we included two as yet undescribed species (N. cf. stygius, N. cf. thienemanni; see also Fišer et al. 2017). The species complex of *N. rhenorhodanensis*, however, containing at least four species is not further resolved and is treated at the complex level.

Easily visible and unambiguous characters were preferentially selected, such as the number and type of setae on maxillae, telson, gnathopod and pereopod dactyls, as well as the urosoma. In addition, characters describing shapes, such as epimeral plates, shapes and size ratios of carpus and propodus of gnathopods, and shapes of coxal plate IV were used. Two characters describe sexual dimorphism, namely the different elongation of rami of uropods. The file is freely available on the website of World Amphipoda Database, and can be used for generating species descriptions, dichotomous identification keys and interactive identification keys (Coleman et al. 2010). The virtue of this file is that anyone can assess their own samples for all traits, add additional taxa and further characters.

## Conclusions

An early attempt of web-initiated collaborative taxonomic research that would foster local taxonomy of *Niphargus* within a unified framework (Fišer et al. 2009b), was only moderately successful (available via http://niphargus.info/morpho-database/). The reasons are unclear, but likely a combination of an insufficient coverage of local faunas and the size of the matrix (high number of characters and many taxa) were not appealing to potential collaborators. By contrast, smaller initiatives, such as presented in this study, seem to be more manageable and in the long run more fruitful. The two DELTA morpho-databases from Middle East and Switzerland are not complete but provide the best overview of the state of *Niphargus* taxonomy in two geographically well-defined regions, and hold promise to stimulate further research in the genus. Both databases are backed with diagnostic sequences. They both enclose a small fraction of morphological variation (19 and 30 characters respectively), making them relatively simple to use. Although each of the two databases contains only a smaller number of species, they jointly present 11 % of all known *Niphargus* species. The virtue of such small, independent studies limited to specific geographic regions is their immediate availability for non-taxonomists. As the number of such revisions increases over time, they could sum into a comprehensive revision of the entire genus.

Our recent work (Altermatt et al. 2014, Fišer et al. 2017) significantly advanced the knowledge on *Niphargus* in Switzerland, and it seems that new findings, such as of *N. aquilex*, are getting rarer. However, the revision of the genus for that area is still incomplete. There are still several open taxonomic questions, including the complex *N. rhenorhodanensis* (Lefébure et al. 2007), as yet formally unnamed lineages that have been provisionally named N. cf. stygius and N. cf. thienemanni (Fišer et al. 2017), and new findings of *N. aquilex* (Trontelj et al. 2009, McInerney et al. 2014). For these species we could not yet draw final taxonomic conclusions with the data at hand, and they require further detailed analyses. Nevertheless, end-users can already combine morphological data and COI sequences and at least identify the main species complexes in Switzerland. We are optimistic this initiative will foster further taxonomic research and, in the near future, close the Swiss chapter of *Niphargus*.

## Supplementary Material

XML Treatment for Niphargus tonywhitteni

XML Treatment for Niphargus luchoffmanni

## Supplementary materials

Supplementary material 1 Table S1. List of samples used in phylogenetic analyses along with GenBank Accessions Data type: species data This is an open access article distributed under the terms of the Creative Commons Attribution License (CC BY 4.0), which permits unrestricted use, distribution, and reproduction in any medium, provided the original author and source are credited.Cene Fišer, Roman Alther, Valerija Zakšek, Špela Borko, Andreas Fuchs, Florian Altermatt

Supplementary material 2 Table S2. List of samples used in a detailed network analyses along with GenBank Accessions Data type: species data This is an open access article distributed under the terms of the Creative Commons Attribution License (CC BY 4.0), which permits unrestricted use, distribution, and reproduction in any medium, provided the original author and source are credited.Cene Fišer, Roman Alther, Valerija Zakšek, Špela Borko, Andreas Fuchs, Florian Altermatt

Supplementary material 3 Table S3. List of new records of Niphargus from Switzerland Data type: occurence This is an open access article distributed under the terms of the Creative Commons Attribution License (CC BY 4.0), which permits unrestricted use, distribution, and reproduction in any medium, provided the original author and source are credited.Cene Fišer, Roman Alther, Valerija Zakšek, Špela Borko, Andreas Fuchs, Florian Altermatt

Supplementary material 4 Morphological database in DELTA Data type: molecular data This is an open access article distributed under the terms of the Creative Commons Attribution License (CC BY 4.0), which permits unrestricted use, distribution, and reproduction in any medium, provided the original author and source are credited.Cene Fišer, Roman Alther, Valerija Zakšek, Špela Borko, Andreas Fuchs, Florian Altermatt
